# Unmasking the Epigenome: Insights into Testicular Cell Dynamics and Reproductive Function

**DOI:** 10.3390/ijms26157305

**Published:** 2025-07-28

**Authors:** Shabana Anjum, Yamna Khurshid, Stefan S. Du Plessis, Temidayo S. Omolaoye

**Affiliations:** College of Medicine, Mohammed Bin Rashid University of Medicine and Health Sciences, Dubai Health, Dubai 505055, United Arab Emirates; shabana.anjum@dubaihealth.ae (S.A.); yamna.khurshid@dubaihealth.ae (Y.K.); stefan.duplessis@dubaihealth.ae (S.S.D.P.)

**Keywords:** epigenetics, spermatogenesis, Leydig cell, Sertoli cell, male infertility, DNA methylation, histone modification, noncoding RNA, transgenerational inheritance

## Abstract

The epigenetic landscape plays a pivotal role in regulating the functions of both germ and somatic cells (Sertoli and Leydig cells) within the testis, which are essential for male fertility. While somatic cells support germ cell maturation and testosterone synthesis, the epigenetic regulation of germ cells is critical for proper spermatogenesis and function. Epigenetic modifications such as DNA methylation, histone modifications, chromatin remodeling, and non-coding RNAs (ncRNAs) are crucial for regulating gene expression that is essential for spermatogenesis and reproductive function. Although numerous studies have highlighted the significance of the epigenome and its implications for male reproductive health, a comprehensive overview of the existing literature and knowledge is lacking. This review aims to provide an in-depth analysis of the role of epigenetics in spermatogenesis and reproductive health, with a specific focus on DNA methylation, histone remodeling, and small noncoding RNAs (sncRNAs). Additionally, we examine the impact of lifestyle and environmental factors, such as diet, smoking, physical activity, and exposure to endocrine-disrupting chemicals, on the sperm epigenome. We emphasize how these factors influence fertility, embryonic development, and potential transgenerational inheritance. This review underscores how recent advances in the understanding of the epigenetic modulation of testicular function can inform the pathophysiology of male infertility, thereby paving the way for the development of targeted diagnostic and therapeutic strategies.

## 1. Introduction

Fertility critically depends on the coordinated interactions between the somatic and germ cells in the testis, which are vital for maintaining its functional integrity. The testicular somatic cells play a specific role in regulating germ cell development and supporting overall testicular function. Sertoli cells provide structural and nutritional support to germ cells, facilitate their maturation, and maintain the blood-testis barrier to protect developing sperm from immune system attacks [1]. Leydig cells, found in the interstitium of the testis, produce testosterone, a key hormone necessary for spermatogenesis and the development of secondary male characteristics. Peritubular (myoid) cells help maintain the structural integrity of the seminiferous tubules by contracting to aid the movement of developing germ cells [2,3]. Germ cells have a unique structure and function, playing a crucial role in fertilization while ensuring the accurate transmission of genetic and epigenetic information to future generations [4,5]. The organization of chromatin within these cells, dependent on complex interactions among DNA, histones, and regulatory molecules, is fundamental to gene regulation during spermatogenesis [6]. Through these interactions, epigenetic marks are established that influence gene expression without altering the DNA sequence. They are heritable and pivotal for sperm development, maturation, and function [7]. Unlike genetic mutations, epigenetic changes do not modify the DNA sequence and can be transmitted across generations, affecting gene expression patterns [8]. Among the key epigenetic marks are DNA methylation, histone modifications, and small non-coding RNAs (sncRNAs), which collectively regulate chromatin structure and gene expression [9]. The concept of epigenetics, initially introduced by Waddington in 1942, is paramount in sperm maturation. This process is dynamically established and erased during spermatogenesis and is essential for controlling gene activity [10,11,12]. In sperm, DNA is highly condensed through protamine-mediated chromatin packaging, protecting the paternal genome during transfer to the oocyte [11,12]. Prior to full maturation, sperm undergo extensive epigenetic reprogramming, which regulates gene expression to maintain genomic integrity [13,14]. Disruptions in these epigenetic processes are increasingly recognized as significant contributors to male infertility because errors in DNA methylation, histone modifications, or sncRNA function can lead to abnormal sperm development [15,16,17,18,19,20,21,22], and many cases of unexplained infertility are now linked to such defects [23,24]. The DNA methylation process often involves the addition of a methyl group to cytosine, which typically results in gene silencing. These unique methylation patterns in sperm have been correlated with neurodevelopmental disorders, metabolic diseases, and risks to transgenerational health [19]. Following fertilization, sperm methylation patterns undergo rapid remodeling, making them highly susceptible to environmental influences. Disruptions in this remodeling process have been associated with embryonic arrest, implantation failure, and developmental disorders [19]. Histones, essential for organizing DNA within sperm, also undergo modifications that regulate gene expression in the developing embryo [13,14]. Moreover, sncRNAs are pivotal for the regulation of gene transcription and translation, influencing both early embryonic development and transgenerational inheritance [19]. The aim of this review is to explore the dynamic role of epigenetic modifications in spermatogenesis, focusing on the significance of DNA methylation, histone retention, and sncRNAs in germ and somatic cell function, embryonic development, and offspring health. Despite substantial advancements in this field, many of the epigenetic mechanisms underlying spermatogenesis remain poorly explored and understood.

## 2. Epigenetic Regulation in Germ Cells

In recent years, epigenetics has become a crucial field that enhances traditional genetic research by exploring gene regulation without altering DNA sequences. Key mechanisms involved include DNA methylation, histone modifications, and microRNA (miRNA) actions, which collectively influence gene expression and cellular development [25,26]. Epigenetics addresses fundamental questions about gene regulation, chromosomal inactivation, and cellular differentiation through processes like chromatin remodeling and RNA interference [27]. Despite significant advancements, many aspects of epigenetic regulation remain poorly understood. Epigenetic reprogramming in the germline is a tightly controlled process that commences early in embryonic development, following a sex-specific pathway [28,29]. In males, primordial germ cells (PGCs) migrate to the genital ridge around the sixth week of gestation, where they interact with somatic cells to form gonads capable of spermatogenesis [30,31]. This intricate process involves mitotic and meiotic divisions and requires precise regulatory mechanisms to maintain genomic integrity and fertility [32].

During spermatogenesis, PGCs undergo several mitotic divisions, maturing into spermatogonia, which can be classified into type A and type B [33]. Type A spermatogonia are stem cells sustaining the pool of germ cells, while type B spermatogonia commit to differentiation, ultimately becoming primary spermatocytes. These primary spermatocytes undergo meiosis to yield secondary spermatocytes, which further develop into spermatids. The final phase, spermiogenesis, involves extensive cellular remodeling that results in mature spermatozoa [34,35]. Throughout these stages, epigenetic modifications play a vital role in regulating gene expression and optimizing post-fertilization development [36,37]. A hallmark of spermiogenesis is the extreme condensation of DNA, facilitated by chromatin remodeling, wherein histones are replaced by nuclear proteins like transition proteins and protamines, allowing for a highly compact sperm chromatin structure [38,39]. Although we have made substantial progress in understanding chromatin remodeling during sperm development, the detailed mechanisms involved remain unclear. DNA methylation serves as another critical component of germline epigenetic reprogramming [28]. During early embryogenesis, global DNA demethylation occurs, restoring totipotency and allowing for unrestricted developmental potential [40]. However, imprinted genes and repetitive sequences retain their methylation patterns, which are essential for normal post-implantation development [41]. De novo methylation is reinstated during the blastocyst stage through the action of DNA methyltransferases (DNMT3a and DNMT3b), while DNMT1 preserves these methylation patterns [42]. Before PGC migration to the genital ridge, extensive epigenetic reprogramming occurs, which facilitates the removal of pre-existing methylation marks and creates a conducive environment for the establishment of sex-specific epigenetic patterns [32,36,43]. Histone modifications represent an additional important layer of epigenetic regulation. In mice, PGCs lose the H3K9me2 modification and acquire H3K27me3 by the end of the first week, which influences gene expression during germ cell differentiation [44,45]. Throughout spermatogenesis, histone tail acetylation plays a significant role as it facilitates the progressive replacement of histones with sperm-specific chromatin proteins. This transition occurs systematically: first, histones are substituted with testis-specific variants, followed by transition proteins and finally protamines, resulting in nearly complete transcriptional silencing of the sperm genome [46]. Notably, the dysregulation of histone modifications can lead to severe consequences, such as impaired spermatogenesis, germ cell apoptosis, and male infertility [47]. Recent studies have identified a unique pattern of H3 methylation in male germ cells, showcasing intricate interactions between H3K4me3 and DNA methylation during spermatogenesis [48,49,50,51]. Collectively, these findings underscore the intricate role of epigenetic modifications in male germline development. Understanding these processes is essential for advancing reproductive biology and may provide insights into infertility, transgenerational epigenetic inheritance, and potential therapeutic interventions for epigenetic disorders [52].

## 3. Epigenetic Regulation of Spermatogonial Stem Cells (SSCs)

**Figure 1:**
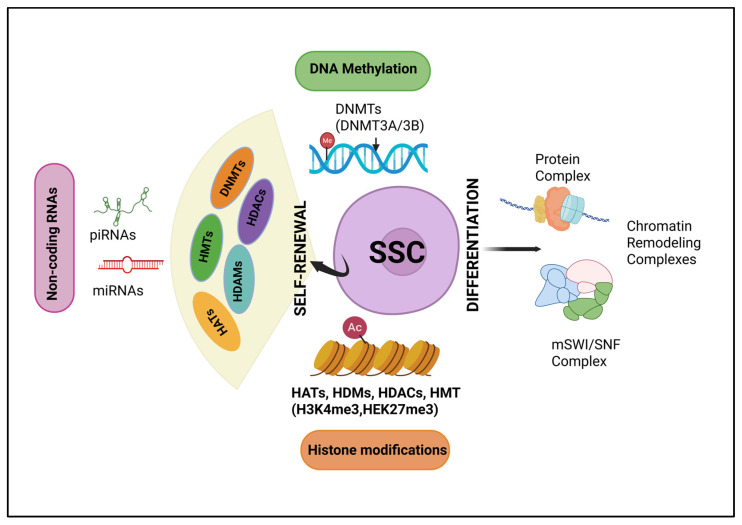
Epigenetic regulation of spermatogonial stem cells: The transition of spermatogonial stem cells (SSCs) into spermatozoa is governed by the complex orchestration of regulatory mechanisms. This transition includes the commencement of meiotic processes and a temporary arrest in the cell cycle. Mechanical and hormonal signals influence signaling pathways that either activate or inhibit histone modification enzymes. DNMTs—(DNA methyltransferases), HATs—(histone acetyltransferases), HDMs (histone demethylases), HMTs (histone methyltransferases), and HDACs (histone deacetylases).

Spermatogonial stem cells (SSCs) reside in the testes of male mammals, where they serve as progenitor cells for spermatogenesis [53]. They are crucial for the continuous production of sperm and are fundamental to maintaining male fertility. In order to maintain the sperm reserves and avoid infertility, surviving SSCs must undergo self-renewal and self-modulation to maintain the SSC pool (Figure 1) [53,54]. Furthermore, spermatogonia undergoes widespread epigenetic remodeling during their differentiation into meiotic spermatocytes [55]. Subsequently, the changes in histone modifications in these spermatogonia will change the expression of transcription factors and adhesion molecules, facilitating the cell to adapt to a new environment [56,57]. At the single-cell level, epigenetic changes can be manipulated according to external signals, influencing cell differentiation or the establishment of cell fate [58,59]. The successful establishment of an interactive microenvironment suggests that these cells have likely embarked on the path of differentiation into spermatogonial cells. Mechanical and hormonal signals often trigger signaling pathways that alter the gene methylation states by either activating or inhibiting the histone methylation (HM) enzymes, i.e., histone methyltransferase (HMTs), histone demethylase (HDMs), which in turn causes the expression of different cell adhesion molecules [56,60]. Ultimately, SSCs adhere to the basement membrane of the seminiferous tubules, where they interact with Sertoli cells and activate their transformation into the differentiating spermatogonial cells (DSCs) and initiate spermatogenesis [57,61]. As a fundamental genomic modification, the dynamic expression of adhesion molecules is regulated by DNA methylation. The progression of SSCs into spermatozoa, including the initiation of meiotic processes and temporary cell-cycle arrest, requires coordination of these intricate regulatory mechanisms [60,62]. Importantly, the differentiation of SSCs to advanced spermatogonial cells cannot be accomplished without proper histone regulations. The dedifferentiation of Dazl-knockout differentiated spermatogonia back into SSCs suggests that spermatogonial cell fate is plastic. Thus, strategies may be employed to reprogram DSCs back to SSCs, thereby creating a novel therapy for male infertility [60].

## 4. Dynamic Epigenetic Regulation of Spermatogenesis

Spermatogenesis is a complex biological process characterized by precise gene regulation, during which testicular germ cells undergo both symmetric and asymmetric divisions to form mature spermatozoa [60,61,62,63]. This development is tightly controlled by epigenetic mechanisms that regulate the spatial and temporal expression of genes essential for lineage-specific differentiation [64,65]. Epigenetic modifications, such as DNA methylation and histone modifications, are crucial for maintaining germ cell self-renewal and differentiation, ensuring proper spermatogenic progression [12,66,67,68,69,70]. The interaction between epigenetic regulation and somatic cell function is critical for balancing self-renewal and differentiation in germ cells, with potential implications for therapeutic interventions in male infertility [12]. Disruptions in these processes can lead to male infertility and reproductive health issues.

Recent studies provide detailed insights into this regulation. DNA methylation, involving the addition of methyl groups to cytosine bases in CpG dinucleotides, contributes to stable gene silencing or activation [71,72]. Additionally, histone modifications, such as acetylation, phosphorylation, and ubiquitination, regulate chromatin accessibility and transcriptional activity essential for gene expression during germ cell development [24,73]. These modifications are dynamically remodeled across spermatogenic stages, providing an additional layer of regulation for stage-specific gene expression [24,73]. Non-coding RNAs also modulate gene expression through transcriptional and post-transcriptional regulatory networks [74,75]. Emerging research indicates that environmental influences such as dietary methyl donors (e.g., folic acid, vitamin B12), oxidative stress, and exposure to pollutants can induce epigenetic alterations that persist in sperm and are potentially transmitted to offspring, impacting embryonic development and health [76,77,78,79,80]. These epigenetic changes may persist through fertilization, affecting embryonic development and offspring health. Insights into the epigenetic dynamics of testicular cells have revealed the heritability of epigenetic information and its role in gene regulation and cellular memory. These epigenetic modifications influence transcription factors binding at specific gene loci, adding complexity to the regulatory networks governing spermatogenesis. Abnormalities in these networks can distort gene expression patterns, culminating in compromised sperm quality and fertility [19,50,81,82].

**Figure 2:**
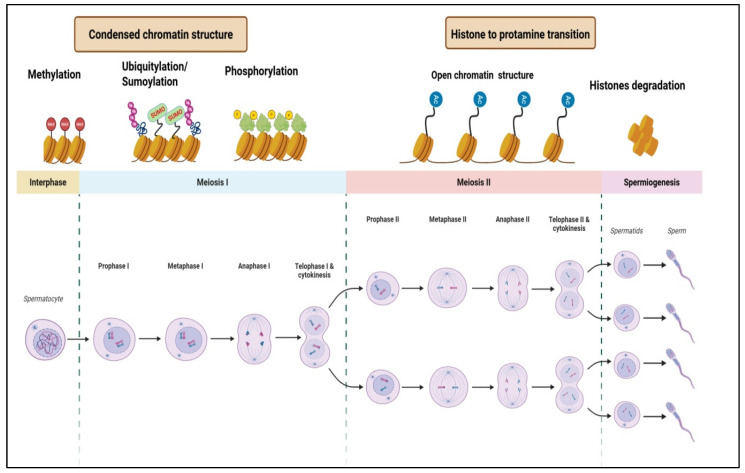
Schematic representation of spermatogenesis and associated epigenetic modifications. The diagram illustrates the stages of spermatogenesis, including mitosis, meiosis I, meiosis II, and spermiogenesis, alongside key epigenetic modifications such as DNA methylation, ubiquitylation, phosphorylation, sumoylation, and histone-to-protamine transition.

The integration of transcriptomic and epigenomic data continues to reveal novel regulatory mechanisms. Recent advances in epigenomic mapping have further clarified how key genes involved in spermatogenic progression undergo stage-specific epigenetic modifications, often targeted by specific epigenetic writers and erasers. These findings underscore the importance of chromatin remodeling in germ cell differentiation and highlight potential therapeutic targets. The integration of transcriptomic and epigenomic approaches continues to reveal novel regulatory mechanisms, offering new avenues for interventions to improve male reproduction. Understanding the dynamic interplay between epigenetic regulation, somatic cell function, and environmental influence, enables researchers to develop targeted interventions to enhance reproductive health. The growing field of epigenetic research not only deepens our understanding of spermatogenesis but also suggests innovative therapeutic strategies in regenerative medicine and infertility treatment. Represented in Figure 2 is a schematic description of spermatogenesis alongside associated epigenetic modifications.

### 4.1. Role of DNA Methylation in Spermatogenesis

Recent studies on testicular cells have increasingly focused on the interactions between germ cells and somatic cells (particularly Sertoli and Leydig cells); these relationships are essential for maintaining spermatogenesis and preserving male fertility. The dynamics of testicular cells undergo significant changes as they progress to a post-mitotic state [83,84,85]. However, our understanding of the epigenetic changes involved is still limited. DNA methylation influences the male germline development, the formation of the testicular niche, spermatogonia maturation, and the differentiation of cells within the testis [13,86]. It is an essential epigenetic mechanism involving the addition of a methyl group to the carbon-5 position of cytosine (5mC), typically in cytosine-based dinucleotides (5′-CpG-3′). This modification can activate or repress gene expression depending on the genomic context [72,81]. In germ cells, cytosine methylation plays a crucial role in preserving genetic integrity and regulating gene expression essential for spermatogenesis [87].

In the testes, CpG sites are hypermethylated during the mitotic division of spermatogonia, playing an important role in deactivating or silencing the irrelevant genes [88,89]. During meiotic division, they become hypomethylated to activate gene expression, enabling spermiogenesis and the formation of haploid sperm cells [87]. DNA methylation and demethylation are primarily mediated by two key gene families, DNA methyltransferases (DNMT3A and DNMT3B) and TET enzymes, respectively, playing crucial roles in both embryogenesis and spermatogenesis [90,91,92]. DNMT3A and DNMT3B establish stage-specific methylation landscapes and silence transposable elements. These are enzymes that add a methyl group to the C5 position of cytosine in CpG dinucleotides, i.e., called de novo methylation [93,94,95]. DNMT1 maintains existing methylation patterns during DNA replication by targeting hemi-methylated DNA strands [95,96,97]. For demethylation, TET enzymes (TET1/2/3), which belong to the Fe(II)/α-ketoglutarate-dependent dioxygenase family, convert methylated cytosines (5mC) into oxidized forms such as 5-hydroxymethylcytosine (hm5C), 5-formylcytosine (f5C), and 5-carboxylcytosine (ca5C). These modified bases are subsequently removed and replaced through the base excision repair (BER) pathway, restoring the unmodified cytosine and effectively erasing the methylation mark. [98]. The disruption of DNA methylation pathways has been closely linked to impaired spermatogenesis and reduced sperm quality, ultimately contributing to male infertility. Studies have demonstrated that knockout of key DNA methylation enzymes leads to defective sperm development, while mutations in DNMT1 in humans result in dysregulated germline methylation associated with idiopathic reproductive failure [81,94,95]. Additionally, abnormal methylation of imprinted genes, particularly following assisted reproductive technologies, can interfere with gene expression and further reduce fertility [99]. A recent study demonstrated the presence of a specific panel of altered gene expression and DNA methylation associated with non-obstructive azoospermia in human testicular meiotic germ cells [100]. These findings highlight the critical role of proper epigenetic regulation in maintaining male reproductive health.

During spermatogenesis, specific methylation patterns at gene promoters, such as *H19* and *DAZL*, undergo dynamic and stage-specific changes. Hypomethylation of *H19* promotes gene activation for germ cell proliferation, while hypermethylation of the *DAZL* gene expression is needed for germ cell maturation [62,101,102,103] Environmental factors like oxidative stress or pollutants can disrupt these patterns, impairing spermatogenesis and contributing to male infertility [80,104,105,106,107]. In mature sperm, important methylation marks are preserved at imprinted loci and precisely regulated. These epigenetic marks are important for proper fertilization and the initiation of early embryonic development. Thus, epigenetic regulation at each developmental stage is essential for normal sperm function and fertility.

### 4.2. Role of Histone Modifications in Spermatogenesis

The modulation of gene expression plays a crucial role in spermatogenesis. The silencing and activation of key genes regulate the differentiation and maturation of both germ cells and somatic cells (Sertoli and Leydig cells) in the testis. In mammals, two germline-dependent cycles of seminiferous epithelium renewal occur after birth. However, in humans, spermatogenic cell division and maturation persist throughout life after puberty [108]. An important epigenetic regulatory network maintains the balance between cell self-renewal, proliferation, and differentiation, ensuring harmonious organism development [109]. Various epigenetic mechanisms regulate testicular cells, including DNA methylation, histone modifications, and the transcription of non-coding RNAs [110]. Among these, histone modifications play a significant role in regulating gene expression [73,110]. Genomic DNA forms chromatin with histones, where the basic unit, the nucleosome, consists of 147 base pairs of DNA wrapped around a histone octamer made up of two copies each of core histones H2A, H2B, H3, and H4 [111,112,113,114]. Histone modifications can influence gene expression by altering chromatin structure, positively or negatively impacting gene accessibility and transcription activation. This process often occurs alongside varying levels of cytosine demethylation [27,115,116]. Modifications to histones are reversible, with histone-modifying proteins able to relocate within the cell, allowing these modifications to be recognized and removed by specific reading proteins [73]. Dynamic histone modifications in response to microenvironmental signals enable more nuanced regulation of gene expression compared to a static chromatin structure [117]. Aberrant histone modifications can be epigenetically inherited, potentially disrupting normal developmental processes [118]. Notably, a significant proportion of males with Down syndrome do not undergo puberty, a phenomenon associated with reduced levels of a key chaperone protein that guides histone-modifying complexes across the genome [119,120]. Additionally, oocytes containing a high abundance of histone variant-containing nucleosomes have been linked to the production of offspring with enhanced developmental robustness, particularly in females [121,122].

### 4.3. Role of Non-Coding RNA (miRNA and lncRNA) in Spermatogenesis:

Recent research has increasingly demonstrated that non-coding RNAs (ncRNAs) serve as key regulators at multiple levels of gene expression during spermatogenesis. ncRNAs are classified into small ncRNAs, including microRNAs (miRNAs) and Piwi-interacting RNAs (piRNAs), and long ncRNAs (lncRNAs) exceeding 200 nucleotides. These molecules play significant roles in post-transcriptional regulation, influencing various stages of germ cell development [123,124,125,126].

MicroRNAs (miRNAs) are conserved, approximately 20–24-nucleotide RNA molecules that regulate protein production after transcription. Early studies suggested that miRNAs and their biogenesis proteins are downregulated during the later stages of spermatid elongation, implying limited roles in sperm development. However, subsequent findings revealed that specific miRNAs, such as miR-34c and miR-138, are involved in critical processes like chromatin remodeling during meiosis and cell cycle regulation. For instance, miR-34c expression increases during spermatogonia entering meiosis and targets genes required for chromatin structure [127,128,129].

Studies have shown that miRNAs are differentially expressed at various stages from spermatogonia to mature sperm, indicating their role throughout germ cell development [130,131]. The deletion of key miRNA-processing enzymes like Dicer and Drosha results in male infertility, which is characterized by defective sperm formation. This study confirms the critical involvement of miRNAs in maintaining fertility [123]. Specific miRNAs also regulate SSCs self-renewal and proliferation during early spermatogenesis, targeting pathways involving STAT3, cyclin D1, Sirt1, and apoptosis mechanisms [132,133,134].

During meiosis, miRNAs such as miR-10a and miR-871 influence homologous recombination and spermatocyte differentiation by targeting genes like RAD51 and Fzd4 [135,136]. Others, including miR-34 b-5p and miR-34c, modulate key cell cycle regulators (e.g., Cdk6, Notch1, MYC), ensuring proper progression through meiotic stages and germ cell maturation [127,128]. MiR-34c contributes to the removal of defective germ cells through apoptosis, serving a quality control function [128].

Long ncRNAs (lncRNAs) have emerged as vital regulators during spermatogenesis, primarily by modulating gene expression through chromatin interactions, transcriptional regulation, and post-transcriptional processes [137,138]. They are expressed in a stage-specific manner, interacting with chromatin-modifying complexes such as Polycomb groups or histone acetyltransferases to activate or repress target genes as needed. The lncRNAs act as molecular scaffolds and facilitate the assembly of transcriptional and epigenetic complexes that ensure precise gene regulation during germ cell development [139,140,141]. Although more research into the specific functions of lncRNAs is ongoing, their importance in supporting spermatogenesis and maintaining male fertility is also being explored.

Overall, ncRNAs are dynamically regulated throughout spermatogenesis and play pivotal roles at multiple stages, from germ cell proliferation to maturation. Advancing our understanding of their mechanisms may reveal novel insights into male fertility and potential therapeutic targets for infertility.

## 5. Epigenetic Modifications in Testicular Somatic Cells

**Figure 3:**
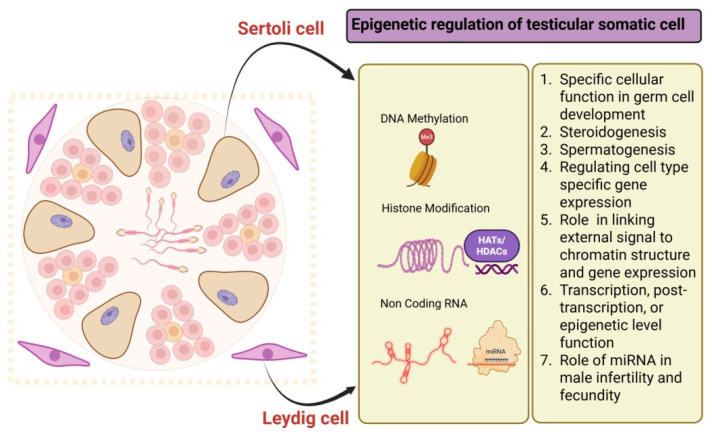
Epigenetic modification in testicular somatic cells (Sertoli cells and Leydig cells).

The testis is a specialized organ responsible for sperm production and hormone regulation, governed by complex epigenetic mechanisms [86]. Each round of spermatogenesis involves significant remodeling of germ cells, orchestrated by somatic cells [142]. Although germline epigenetics has received greater attention, somatic cells such as Sertoli, Leydig, and peritubular myoid cells also undergo epigenetic modifications that are essential for supporting spermatogenesis and maintaining overall testicular function (Figure 3). Sertoli cells, developed from nuclear budding in somatic cells, facilitate the polarization and migration of germ cells [143]. These modifications establish a unique histone code pattern, essential for the tightly regulated gene network involved in sperm development. Research has focused on the diurnal oscillation of acetylation and phosphorylation [144], with key factors including DNA methylation, histone modifications, and non-coding RNA-mediated regulation, all influencing gene expression vital for testicular development, steroidogenesis, and spermatogenesis [144]. Before birth, maternal cues transcriptionally activate several testis-specific genes, such as *SRY*, *SOX9*, *AMH*, *TDF*, and *SF1*, in the somatic cells of the developing testes [145,146,147]. Dysregulation of these modifications is linked to various diseases, including infertility [148,149].

### 5.1. Role of DNA Methylation in Testicular Somatic Cells

Somatic cells play a fundamental role in germ cell development and the structural organization of the testis [150]. Gaining a comprehensive understanding of the molecular pathways that drive the progression from immature precursor cells to fully differentiated somatic cell types is essential. These findings not only elucidate the sequential nature of these developmental processes but also highlight potential molecular targets for therapeutic intervention in disorders such as testicular dysgenesis syndrome and certain forms of male infertility associated with somatic cell dysfunction in the testis [151]. It is evident that epigenetic regulation of gene expression is a key determinant in cellular differentiation and guiding cells through the intricate development of multicellular organisms [152]. However, it is important for the stability of cellular identity throughout an organism’s lifespan. Recent research has mapped DNA methylation dynamics during postnatal testicular development, revealing significant shifts in one of the most extensively studied epigenetic modifications in vertebrates [153,154,155,156]. This extensive dataset offers valuable insights into how dramatic epigenetic reprogramming during critical developmental transitions influences gene expression patterns, particularly in Sertoli and Leydig cell maturation. Evidence suggests that developmentally regulated methylation patterns play a pivotal role in fine-tuning the gene regulatory network essential for the functional differentiation of these somatic cell populations [157,158]. Additionally, specific alterations in the methylation landscape have been linked to cellular commitment, reinforcing the notion that DNA methylation is integral to maintaining and transmitting stable cellular identities across generations [159,160,161]. Furthermore, the translational relevance of these naturally occurring epigenetic mechanisms is recognized, particularly in the context of environmental influences that may disrupt regulatory networks [70,162]. Emerging studies suggest that external factors can interfere with these epigenetic safeguards, potentially compromising developmental stability [162]. While the clinical implications of these findings are not the primary focus, they are significant given the alarming rise in male fertility issues observed in recent years. Understanding how epigenetic mechanisms regulate somatic cell function in the testis could pave the way for novel therapeutic approaches to address infertility and related disorders [70]. The dynamic nature of DNA methylation during the maturation of testicular somatic cells is highlighted by two key processes. First, during embryonic development, DNA methylation is removed from somatic cell progenitors and later re-established in a tissue-specific manner after birth [163]. Second, maintaining DNA methylation patterns is essential for keeping mature somatic cells specialized and preventing defects that could lead to diseases such as testicular cancer and infertility [82]. Abnormalities in DNA methylation patterns are often associated with reproductive issues, including male infertility.

### 5.2. Role of Histone Modification in Testicular Somatic Cells

The cell-type-specific epigenetic landscape is shaped by various post-translational histone modifications [60,164,165,166,167]. The dynamic changes in histone modifications during testicular development and somatic cell maturation are critical for regulating cell-type-specific gene expression. However, the investigation of histone modifications specific to somatic cells has been limited. These modifications are regulated by histone-modifying enzymes, such as HATs and HDACs, which play a crucial role in translating external signals into chromatin structure and gene expression [166,167]. These modifications determine functional outcomes such as transcriptional activation or repression by coordinating a multi-protein network that adjusts chromatin structure, thereby influencing accessibility to regulatory genome regions [168,169]. The interaction between transcription factors and target DNA sequences influences global histone modification patterns and the gene expression profile of specific cell types [115,170]. Previous studies have shown that alterations in histone marks are linked to distinct cellular functions in the rat testis, where testicular somatic cells play a vital role in germ cell development [31,49,171,172]. Research indicates that changes in histone marks can alter chromatin remodeling, subsequently affecting the transcriptome and cellular maturation in the testis [173]. Using rodent models, studies have systematically explored how key histone modifications regulate gene expression related to the cell cycle, transcription factors, and the steroidogenic pathway, examining the effects of histone marks on chromatin structure at a genome-wide level [174,175,176,177,178]. Findings reveal that H3K4me3 and H3K27me3 are enriched at promoter regions, affecting adjacent gene expression and correlating with specific chromatin structures. Additionally, some of these modifications are conserved and linked to cellular maturation in both rat and mouse testes [171,179,180,181]. External factors, including testosterone and luteinizing hormone, can also modify histone marks and influence Sertoli and Leydig cell functions. Research shows that these hormones regulate specific histone changes with age in the rat testis, which correlate with alterations in genes related to cell development and function [147,182]. Transcriptome analyses indicate that aging affects several genes involved in testosterone and luteinizing hormone signaling, which are associated with changes in repressive histone marks like H3K27me3 [147,183]. Utilizing global genomic methods to study histone modifications offers valuable insight into how these changes occur at a genome-wide level during somatic cell development in rats. Investigating histone modifications in testicular cells will enhance our understanding of the complex gene regulation networks governing the maturation of testicular somatic cells. A histone acetyltransferase, p300, activates SOX9 transcription in Sertoli cells through histone acetylation at enhancer regions, confirming its role in male differentiation [184]. These findings, supported by broader insights from Kumar and Roy (2024), demonstrate that the coordination of DNA and histone modifications is critical for regulating sex-determining genes and preventing disorders of sexual development [185]. The histone acetyltransferases CBP (CREBBP) and p300 (EP300) regulate gene expression by modifying chromatin. This study shows that both enzymes are required for sex determination in mice. Deleting either gene in embryonic gonadal somatic cells leads to abnormal XY gonad development and partial or complete sex reversal, depending on the number of functional alleles. Mutant gonads exhibit reduced expression of Sry and Sox9, key testis-determining genes, along with lower H3K27ac levels at the Sry promoter. These findings highlight CBP/p300’s role in epigenetically activating Sry and suggest potential links to human disorders of sex development (DSD) [186].

### 5.3. Role of Non-Coding RNAs (miRNA and lncRNA) in Testicular Somatic Cells

The maturation of testicular somatic cells is crucial for male fertility and is influenced by multiple regulatory layers. In Sertoli cells, cytokines and androgens interact to modulate gene expression through a paracrine signaling network, alongside intrinsic genetic factors like long non-coding RNAs (lncRNAs) [187]. In Leydig cells, lipid metabolism and steroidogenesis are regulated by hormonal signaling pathways and the cellular environment, with miRNAs directly influencing key genes [188]. Non-coding RNAs (ncRNAs) serve as vital post-transcriptional regulators, acting at various levels, including transcriptional, post-transcriptional, and epigenetic [189]. The non-coding RNAs (ncRNAs) encompass small RNAs, such as microRNAs (miRNAs) and long non-coding RNAs (lncRNAs of over 200 bp. miRNAs are a well-studied type of ncRNA [190], with certain testis-specific miRNAs linked to male fertility and fecundity [191]. The lncRNAs, which are long RNA transcripts without significant open reading frames, can influence various activities of the gene transcription machinery [192]. They are involved in processes like steroidogenesis, epithelial-to-mesenchymal transition, tight junctions, and lipid metabolism [165]. Changes in lncRNA expression in somatic cells may contribute to animal subfertility when these cells are deficient [137,138,193]. Recent research indicates that environmental factors can impact lncRNA expression and may exert a programming effect. A recent study has shown that prenatal under-nutrition induces changes in lncRNA expression in postnatal male reproductive organs, along with alterations in methylation at promoter sites [194]. Exposure to environmental chemicals during the neonatal period alters ncRNA profiles in the rat testis during puberty, disrupting normal development and suggesting epigenetic implications [195].

There is a growing interest in understanding the timing of lncRNA transcription, particularly testis-specific lncRNAs, and their roles in reproduction-related processes and disorders. This knowledge could enhance the use of lncRNAs as diagnostic or prognostic markers and therapeutic targets. Given the established role of ncRNA regulation in controlling Sertoli and Leydig cell development and function, understanding how spatial and temporal variations in ncRNA profiles affect these cells is vital for fertility and testicular health.

## 6. Impact of Environmental Factors and Lifestyle on Epigenetic Changes in Sperm

**Figure 4:**
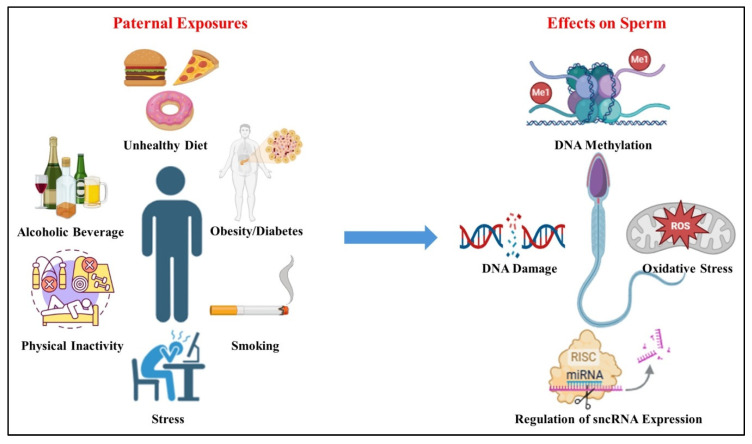
Paternal exposures and their effect on sperm. Paternal exposures such as unhealthy diet, alcoholic beverage consumption, physical inactivity, obesity/diabetes, smoking, and stress can affect sperm epigenetic factors. These factors include DNA methylation; increased DNA damage in sperm, potentially leading to mutations and impaired fertilization; oxidative stress; elevated levels of reactive oxygen species (ROS) causing oxidative damage to sperm cells and their DNA; regulation of sncRNA expression; and disruption of small non-coding RNA (sncRNA) expression, which plays a role in regulating gene expression during spermatogenesis and early embryonic development.

Sperm health and fertility are significantly affected by lifestyle and environmental factors, including diet, smoking, physical activity, and exposure to endocrine-disrupting chemicals (EDCs) (Figure 4) [77,196,197,198,199,200]. These elements are crucial for maintaining a healthy lifestyle and ensuring the epigenetic mechanisms that regulate spermatogenesis and support viable pregnancies [77,107,200]. Environmental agents like bisphenol A, tributyltin, vinclozolin, diethylstilbestrol, methoxychlor, chemotherapy agents, cannabis, flame retardants, mercury, polycyclic aromatic hydrocarbons, phthalates, pesticides, and heavy metals have all been shown to alter sperm DNA methylation profiles, thereby affecting fertility and potentially the health of offspring. These changes involve epigenetic mechanisms such as DNA methylation, histone modification, and disruptions in small RNA pathways [201]. Germ cells are especially vulnerable to EDCs during fetal development, and such exposures may cause transgenerational effects on male fertility through heritable epigenetic changes.

Exposure to industrial pollutants such as heavy metals, solvents, and petroleum-derived compounds can disrupt gene expression involved in spermatogenesis, including conserved genes like *SOHLH2*, *DAZL*, *NANOS2*, *DND1*, *PRDM9*, and *TAF7L*, and can induce DNA damage and epigenetic modifications [202,203]. Notably, even non-genotoxic chemicals capable of crossing the blood-testis barrier pose significant risks. This barrier, formed by Sertoli cells, is selectively permeable to electrolytes, small polar molecules, and some lipophilic substances and plays a vital role in protecting germ cells from xenobiotics. In rodents, epigenetic alterations occur during fetal germ cell development, when the genome undergoes extensive chromatin remodeling [29]. Disruptions during this stage can impair spermatocyte development, leading to spermatogenic arrest, atrophy, and altered sperm parameters [203].

Paternal lifestyle and diet before conception can influence offspring health through epigenetic inheritance, affecting sperm DNA methylation, histone modification, and small non-coding RNA (sncRNA) expression [107]. Smoking has been linked to hypermethylation of genes involved in antioxidant defenses and insulin resistance [77,204,205]. Additionally, paternal diet and obesity raise the risk of metabolic issues in offspring via epigenetic changes in sperm [149,206,207]. Thus, dietary interventions may improve reproductive health. Evidence suggests that diet can impact sperm DNA integrity [208,209,210,211,212]. This study examines the relationship between diet and the epigenetic profile of sperm, highlighting the need for further research to establish dietary guidelines that enhance sperm quality and pregnancy success. Certain dietary components may interact with folates in biological fluids, affecting gene expression, histone methylation, and DNA methylation [48]. These alterations could lead to transcriptional silencing, increasing the risk of fertility-related disorders and developmental issues in offspring [48].

Epigenetic marks in sperm, including DNA methylation, histone modifications, and sncRNA, play a critical role in zygotic genome programming and offspring health [19,50]. Following fertilization, DNA undergoes global demethylation and de novo methylation, but certain loci involved in pluripotency and early embryogenesis remain methylated and escape demethylation [156,213]. Recent findings have shown that some amino acids enhance sperm quality by scavenging reactive oxygen species (ROS), preserving sperm count and motility [78,214,215,216,217,218]. The amino acids, N-acetyl-L-cysteine (NAC), reduce oxidative stress in human semen, potentially improving fertility [219], and alpha-lipoic acid (ALA) exhibits antioxidant properties that may protect sperm DNA and enhance motility [219]. Paternal lifestyle and diet significantly impact offspring health through epigenetic mechanisms, including DNA methylation and histone modification [19,107]. Smoking, obesity, and chronic stress can negatively alter sperm epigenetics, leading to metabolic dysfunction and fertility issues in future generations [213]. Nicotine (major component of smoking) exposure can induce DNA methylation changes in Sertoli and Leydig cells and key genes within the testis via regulating hedgehog pathways [220,221,222]. It increases methylation at the Pebp1 transcription start site, disrupting the ERK pathway and impairing spermatogenesis [223]. Conversely, nicotine-induced hypomethylation of the Pfn1 gene may enhance sperm motility in mice [221]. It also alters histone acetylation, particularly of histone H4, contributing to premature nuclear protein transition and infertility [15]. Moreover, smoking affects the expression of non-coding RNA (miRNAs) in testicular somatic cells, influencing critical regulatory networks in spermatogenesis. It alters the expression of 28 miRNAs, including miR-652 and miR-30c, which are vital for sperm quality and embryo development [224]. These epigenetic changes lead to the disruption of spermatogenesis and ultimately male infertility. Exposure to EDCs further increases disease risks [211,213]. Additionally, paternal factors can affect assisted reproductive technology (ART) outcomes [225]. This study emphasizes the need for further exploration of epigenetic markers in sperm. Understanding how paternal lifestyle and environmental factors impact fertility and offspring health may inform clinical practices designed to encourage healthier behaviors, potentially reversing epigenetic changes in sperm, improving sperm quality, and reducing the risk of metabolic diseases in fathers and future generations.

## 7. Reproductive Health Outcomes and Epigenetic Inheritance

Epigenetic modifications, which influence gene expression by altering DNA and chromatin states, are crucial in both development and disease. Different epigenetic patterns in diseases can serve as biomarkers for diagnosis and monitoring disease progression. Research indicates that men’s lifestyles and environmental factors can cause epigenetic changes in sperm, potentially transmitting changes to offspring and affecting post-fertilization development [77,107,196,199,200]. These modifications can influence sperm development and DNA integrity, with possible transgenerational effects [52,107,226,227]. In the post-genomic era, the study reported that only 1–2% of the human genome codes for proteins; the remaining 98% of the human genome is not yet known [228]. While much of this non-coding genome is poorly understood, some regions have been associated with regulatory activity, contributing to genetic and epigenetic variations linked to diseases.

### 7.1. Role of Epigenetics in Infertility

Male factor infertility accounts for 50% of all infertility cases, and for most patients, the underlying causes remain unknown [229]. Epigenetics plays a crucial role in the regulation of gene expression without altering the DNA sequence itself, and its influence on infertility has garnered significant attention in recent years. Epigenetic mechanisms such as DNA methylation, histone modifications, and non-coding RNAs are essential for proper gametogenesis, fertilization, and early embryonic development [82]. Aberrations in these epigenetic marks can disrupt sperm and oocyte quality, leading to impaired fertilization, embryo development failures, or early pregnancy loss [230,231,232]. During spermiogenesis, the haploid genome of sperm undergoes extensive chromatin remodeling. This results in a highly condensed and stable structure, reduced by more than 5800-fold in volume, to ensure efficient transmission of paternal genetic and epigenetic information at fertilization [226,227,228,229,230,231,232,233]. During spermatogenesis, transfer RNAs (tRNAs) and their fragments, such as small temporal RNAs (stRNAs), are synthesized by RNA Polymerase III (RNAPIII) enzymes. These transcripts are then processed by RNase Z, which removes the 3′ trailer sequences to produce 5′ pre-tRNA halves (30–50 base pairs) and 3′ trailer halves (20–25 base pairs). Emerging evidence suggests that these tRNA-derived fragments (tRFs) play regulatory roles in spermatogenesis, including gene silencing and stress response. Altered expression or biogenesis of tRFs has been associated with impaired sperm function and male infertility, highlighting their potential as epigenetic regulators of reproductive health [234]. Post-transcriptional nucleotide modifications in tRNA, including 5-methyl-cytosine (m5C), have been shown to modulate codon decoding, prevent tRNA cleavage, and be reversible [235]. Abnormal DNA methylation plays a vital role in regulating gene expression in spermatozoa, that linked to spermatogenesis, embryo development, and male fertility [9,101]. When this process is disrupted, it can interfere with sperm development and function, potentially leading to infertility. Studies have shown that many genes linked to male infertility exhibit abnormal DNA methylation patterns. These irregularities can disturb the expression of key genes involved in reproduction, contributing to fertility issues [221,236,237,238,239,240,241,242]. For instance, researchers have found altered methylation in the promoter region of the *methylenetetrahydrofolate reductase* (*MTHFR*) gene, as well as hypomethylation at the *IGF2-H19* locus and hypermethylation in genes like *MEST* and *SNRPN* [221,237].

These findings highlight that infertility is not only a genetic issue but also an epigenetic disorder influenced by environmental factors, lifestyle, and aging, emphasizing the importance of epigenetic research in developing diagnostic and therapeutic strategies for infertility.

### 7.2. Role of Epigenetics in Assisted Reproductive Technologies (ART)

The last four decades have seen remarkable advancements in assisted reproductive technology (ART), facilitating fertilization and early embryo development in vitro. ART is commonly utilized to alleviate fertility problems, but some procedures can introduce additional epigenetic modifications in sperm or enable altered sperm with defective epigenomes to generate embryos [243]. This is attributed to its specific characteristics and the availability of reliable tools to assess both localized and global changes. Studies indicate that ART is associated with epigenetic alterations in both embryonic and extra-embryonic tissues [166,244,245,246,247]. Consequently, it is plausible that the epigenetic modifications resulting from the conditions associated with in vitro fertilization (IVF) play a significant role in the development of some of the adverse outcomes observed after ART [243].

The intracytoplasmic sperm injection (ICSI) technique itself could have a role in inducing epigenetic alterations. During the ICSI procedure, a needle is inserted into the ooplasm to inject a spermatozoon into the oocyte [248]. This physical isolation from the sperm membranous structures, proteins/nucleo-histones bound to DNA, and signals provided by the female reproductive system potentially alter the natural signaling mechanisms underlying sperm activation. A parallel with animal models indicates that ICSI is associated with epigenetic remodeling of histone marks in sperm after fertilization [243]. These changes could affect gene expression patterns in the developing embryo; thus, the use of ICSI in human reproduction would require a thorough re-evaluation of the long-term health outcomes of children conceived this way [150,243,249].

### 7.3. Therapeutic Implications

Epigenetic drugs are designed to modify DNA methylation and histone modifications and are used to treat diseases like cancer and autoimmune disorders [250]. Studies show that certain drugs can alter sperm DNA methylation and influence offspring phenotypes without significantly affecting fertility, highlighting the need for careful consideration of epigenomic drug exposures [251,252]. In the gene therapy approach, after fertilization, the information is transferred to the zygote and ultimately to all cells of the next individual. Thus, germ cells are a potential target for the impact of epigenomic drugs and have the potential to have wide-reaching and long-lasting impacts on health and development. A study by Jarred (2018) shows that epigenomic drugs and germline impacts performed in rodent models alter sperm DNA methylation [106,253]. It also highlights diseases like phenylketonuria, where genes for necessary enzymes can improve treatment outcomes [193].

## 8. Transgenerational Epigenetics and Its Effect on Reproductive Health

Alterations in gene expression or cellular phenotype that are not reliant on changes in the DNA sequence are called epigenetic, although several lines of evidence suggest that the external environment can have an impact on germ cell development and epigenetic marks [254]. Understanding the exact molecular pathways underlying the reprogramming process and inheritance of certain epigenetic states to the offspring is a challenging issue. Studies have shown that significant reprogramming of DNA methylation and histone modifications occurs in gametes and zygotes, which is crucial for initial embryo formation [255,256,257]. Disruptions in this reprogramming can affect fertility, pregnancy, and offspring health, potentially leading to the occurrence of developmental abnormalities and other diseases of adulthood [258]. There is evidence that suggests that environmental factors experienced by parents can induce epigenetic changes, potentially impacting subsequent generations even without direct exposure [77,78,107,196,197,198,199,200,259]. The interplay between genetic and epigenetic factors offers new insights into disease mechanisms and highlights the importance of understanding how environmental exposures shape health outcomes across generations. As epigenetics evolves, it shifts the focus of drug discovery from a purely genetic model to a broader, disease-centric perspective, demonstrating connections between lifestyle, environment, and health. To date, studies have focused on children and grandchildren (F1–F3 generations) to investigate transgenerational epigenetic changes induced by ancestral exposure to certain environmental factors, even in the absence of direct exposure in these later generations [260,261,262,263,264,265,266]. It investigates how environmental factors affect gene expression across generations, revealing the potential for inherited epigenetic changes linked to diseases. Research highlights the complex interplay between genetics and external influences on health outcomes, emphasizing the need for further studies. Reproductive health with epigenetics is playing a role in transgenerational inheritance of traits influenced by environmental factors, affecting gametogenesis and fertility across generations. Understanding these connections is vital for future health management.

## 9. Conclusions and Future Perspectives

The field of epigenetics has transformed our understanding of the molecular regulation of spermatogenesis and male fertility. It is now clear that epigenetic mechanisms such as DNA methylation, histone modifications, and non-coding RNAs play essential roles in germ cell development, sperm maturation, and fertility potential. Aberrant DNA methylation patterns have been consistently associated with impaired sperm function and male infertility, while disruptions in histone modifications affect chromatin structure and gene expression. These epigenetic marks not only influence germ cell differentiation but also impact embryo development, underscoring their significance in reproductive health. Non-coding RNAs, including microRNAs and piRNAs, have emerged as critical regulators of gene expression in spermatogonial stem cells. These molecules guide epigenetic programming during early spermatogenic stages and are involved in maintaining the balance between stem cell renewal and differentiation. Their dysregulation has been linked to spermatogenic failure, reinforcing the complexity of the epigenetic control network in the male germline.

Importantly, environmental factors are now recognized as significant modulators of the epigenome. Exploring epigenetic dynamics in testicular cells reveals the complex interplay between environmental factors and genetic expression, emphasizing the role of epigenetic modifications in male fertility. Studies have demonstrated that exposure to endocrine-disrupting chemicals, heavy metals, and other environmental toxins can induce stable epigenetic changes in the male germline, which may be transmitted to future generations. This transgenerational epigenetic inheritance has profound implications for male fertility and offspring health. Jenkins et al. provided compelling evidence that paternal environmental exposures can alter DNA methylation patterns in sperm and reduce fertility in subsequent generations, highlighting the long-term consequences of such exposures [9]. Despite these advances, many critical questions remain unresolved. The extent to which environmentally induced epigenetic changes are reversible remains unclear, and the development of interventions to correct harmful epigenetic alterations is still in its early stages. Moreover, while associations between specific epigenetic changes and infertility have been reported, establishing direct causal links requires more robust and mechanistic studies. Another major limitation in the field is the lack of standardized, sensitive, and reproducible methods for detecting and quantifying epigenetic modifications, which hinders the comparison of findings across studies.

Future research must aim to fill these gaps by focusing not only on germ cells but also on somatic cells within the testes, such as Sertoli and Leydig cells. These cells provide structural and hormonal support for spermatogenesis and are known to undergo epigenetic changes during testicular development. However, their specific epigenetic roles remain poorly defined and warrant further investigation. Ultimately, understanding how epigenetic modifications contribute to male fertility will open new avenues for diagnosis and treatment. Epigenetic profiling of infertile men may lead to personalized therapeutic strategies and better outcomes in assisted reproductive technologies. Furthermore, public health initiatives aimed at minimizing harmful environmental exposures could help preserve male reproductive health across generations. As the epigenetics field continues to expand, it holds great promise for advancing reproductive medicine and improving fertility management in both clinical and environmental contexts.
